# Inference of Essential Genes of the Parasite *Haemonchus contortus* via Machine Learning

**DOI:** 10.3390/ijms25137015

**Published:** 2024-06-27

**Authors:** Túlio L. Campos, Pasi K. Korhonen, Neil D. Young, Tao Wang, Jiangning Song, Richard Marhoefer, Bill C. H. Chang, Paul M. Selzer, Robin B. Gasser

**Affiliations:** 1 Department of Biosciences, Melbourne Veterinary School, Faculty of Science, The University of Melbourne, Parkville, VIC 3010, Australia; tulio.campos@fiocruz.br (T.L.C.); pasi.korhonen@unimelb.edu.au (P.K.K.); nyoung@unimelb.edu.au (N.D.Y.); tao.wang1@unimelb.edu.au (T.W.); bchang@ozomics.com (B.C.H.C.); 2 Bioinformatics Core Facility, Aggeu Magalhães Institute (Fiocruz), Recife 50740-465, PE, Brazil; 3 Department of Data Science and AI, Faculty of IT, Monash University, Melbourne, VIC 3800, Australia; jiangning.song@monash.edu; 4 Biomedicine Discovery Institute, Department of Biochemistry and Molecular Biology, Monash University, Clayton, VIC 3800, Australia; 5 Monash Data Futures Institute, Monash University, Clayton, VIC 3800, Australia; 6 Boehringer Ingelheim Animal Health, Binger Strasse 173, 55216 Ingelheim am Rhein, Germany; richard.marhoefer@boehringer-ingelheim.com (R.M.); paul.selzer@boehringer-ingelheim.com (P.M.S.)

**Keywords:** *Haemonchus*, *Caenorhabditis*, *Drosophila*, essential genes, prediction/prioritisation, Eukaryota, Ecdysozoa, machine learning

## Abstract

Over the years, comprehensive explorations of the model organisms *Caenorhabditis elegans* (elegant worm) and *Drosophila melanogaster* (vinegar fly) have contributed substantially to our understanding of complex biological processes and pathways in multicellular organisms generally. Extensive functional genomic–phenomic, genomic, transcriptomic, and proteomic data sets have enabled the discovery and characterisation of genes that are crucial for life, called ‘essential genes’. Recently, we investigated the feasibility of inferring essential genes from such data sets using advanced bioinformatics and showed that a machine learning (ML)-based workflow could be used to extract or engineer features from DNA, RNA, protein, and/or cellular data/information to underpin the reliable prediction of essential genes both within and between *C. elegans* and *D. melanogaster*. As these are two distantly related species within the Ecdysozoa, we proposed that this ML approach would be particularly well suited for species that are within the same phylum or evolutionary clade. In the present study, we cross-predicted essential genes within the phylum Nematoda (evolutionary clade V)—between *C. elegans* and the pathogenic parasitic nematode *H. contortus*—and then ranked and prioritised *H. contortus* proteins encoded by these genes as intervention (e.g., drug) target candidates. Using strong, validated predictors, we inferred essential genes of *H. contortus* that are involved predominantly in crucial biological processes/pathways including ribosome biogenesis, translation, RNA binding/processing, and signalling and which are highly transcribed in the germline, somatic gonad precursors, sex myoblasts, vulva cell precursors, various nerve cells, glia, or hypodermis. The findings indicate that this in silico workflow provides a promising avenue to identify and prioritise panels/groups of drug target candidates in parasitic nematodes for experimental validation in vitro and/or in vivo.

## 1. Introduction

Parasitic worms (helminths) cause substantial mortality and morbidity in animals and substantial losses for agriculture and food production globally. Roundworms (nematodes) cause particularly destructive diseases in livestock animals, affecting hundreds of millions of animals (e.g., sheep, goats, cattle, and pigs) worldwide, resulting in substantial economic losses (billions of dollars) per annum globally [1,2,3]. Despite substantial efforts to control gastrointestinal worms, highly effective commercial vaccines are lacking, and treatment relies heavily on only a small number of anthelmintics, such as monepantel, albendazole, and/or ivermectin [4]. Because anthelmintic resistance to the majority of these compounds is now widespread [5,6,7], there is substantial demand for new anthelmintics with mechanisms/modes of action that are distinct from those presently available on the market and to which resistance has developed. 

The discovery of novel anthelmintic targets using conventional methods is time-consuming and challenging [8,9], and we have been promoting and evaluating the use of in silico methods for the prediction and prioritisation of ‘essential genes’ for subsequent validation as drug target candidates. Our initial exploratory studies [10,11] have been focused on assessing and employing machine learning (ML)-based approaches for the prediction of such genes in the most intensively studied multicellular model organisms—*Caenorhabditis elegans* (free-living nematode) and *Drosophila melanogaster* (vinegar fly). We have taken this focus because these organisms can be maintained in cultures in the laboratory and, importantly, because chromosome-continuous genomes and extensive functional genomic, transcriptomic, proteomic, biochemical, physiological, biological, morphological, developmental, and reproductive data sets and information are publicly available via well-curated databases including WormBase and FlyBase [12,13,14,15]. This wealth of resources has enabled deep and meaningful investigations of essential genes [16,17] for these two Ecdysozoan species. Our ML-based studies have shown that informative features can be extracted/engineered from such data sets, allowing the confident (statistically valid) prediction and prioritisation of known essential genes both within and between *C. elegans* and *D. melanogaster* (see [10,11,16,17]). 

As the complete life cycles of species of gastrointestinal nematodes (order Strongylida, or strongylid nematodes) cannot be maintained in vitro, and laboratory culture conditions vary from those in nature (in the environment and in the host animal), establishing functional genomic assays for the different developmental stages and sexes of these parasitic nematodes has been a major obstacle [18,19] to evaluating the essentiality of genes in order to infer or prioritise intervention target candidates. Now that we have demonstrated the feasibility of an ML-based bioinformatic approach for the reliable prediction and prioritisation of essential genes in *C. elegans* and *D. melanogaster*, and in both of these species [16,17], we are confident that this approach can predict essential genes across relatively closely related species of nematodes or arthropods (Ecdysozoa) provided that suitable data sets are available for analyses. For us, the next logical step is to infer such genes in economically important strongylid nematodes, which are in the same evolutionary clade (V) as *C. elegans*, so that the large-scale inference of essential genes across related species becomes feasible and efficient. 

Here, we selected *Haemonchus contortus* (barber’s pole worm) as a key representative of the order Stronglida because it is one of the most pathogenic nematodes of livestock animals worldwide and because it is now one of the best-studied parasitic nematodes at the molecular level, thus being elevated to model organism status [20,21,22]. Importantly, a chromosome-contiguous genome is available for *H. contortus*, and extensive genomic, genetic, transcriptomic, variomic, and proteomic data sets are readily accessible publicly (via WormBase ParaSite; cf. [20,21,23,24]) for comprehensive in silico explorations. 

In this study, we harnessed these data sets for *H. contortus* to predict and prioritise essential genes in this species through ML (cf. [17]), employing data predominantly for *C. elegans* and *D. melanogaster* for algorithm training purposes and comparative analyses, and we explore the relationship between gene essentiality and transcription in *H. contortus*.

## 2. Results

### 2.1. Identification of Strong Predictors of Essential Genes in H. contortus

In total, we extracted or engineered 9588 features for 19,450 protein-coding genes encoded in the nuclear genome of *H. contortus*. Of these features, we selected 25 that had been identified previously as strong predictors of gene essentiality within *C. elegans* and *D. melanogaster* (see [10,11]) and between these two species (see [16]); we also selected two more features: “num_cells_expressed”, the number of nuclei in which individual genes are transcribed in *H. contortus* (eggs), and “evolutionary conservation” among 16 divergent eukaryotic species including *H. contortus*. The descriptions of the 27 features used for the prediction of essential genes are given in the Table in the Materials and Methods section. 

**Figure 1:**
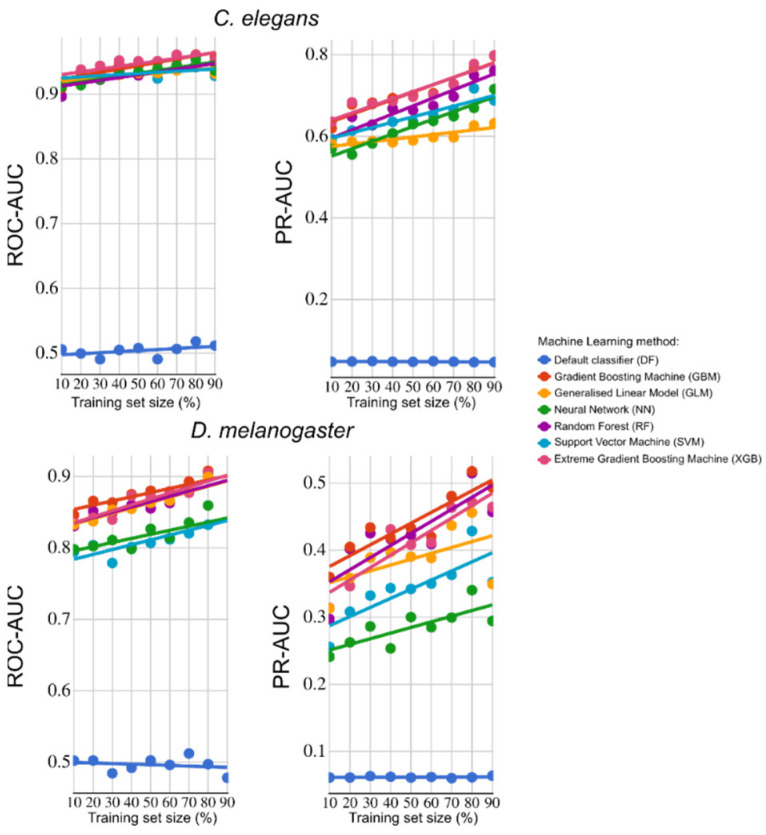
Machine learning (ML) performance metrics (ROC-AUC and PR-AUC) for the prediction of essential genes in *Caenorhabditis elegans* (**top**) or *D. melanogaster* (**bottom**) using features available for *Haemonchus contortus*. ML methods used: Gradient Boosting Machines (GBMs), Generalised Linear models (GLMs), Neural Networks (NNs), Random Forest (RF), Support Vector Machines (SVMs), and Extreme Gradient Boosting Machines (XGBs).

Prior to the prediction of essential genes, we evaluated the predictive power of this set of 27 features for *C. elegans* and for *D. melanogaster* using ML approaches and a subsampling strategy for training, testing, and evaluation (ROC-AUC and PR-AUC metrics). For *C. elegans* (Figure 1), the ROC-AUC value was ≥0.9 for all six ML models assessed (i.e., GBM, GLM, NN, RF, SVM, and XGB). The PR-AUC value was >0.6, achieving close to 0.8 for the best performers (GBM and XGB) using 90% of the data to train the models. For *D. melanogaster* (Figure 1), the ROC-AUC value was ≥0.8, achieving ~0.9 for the best performers (GBM, GLM, RF, and XGB). The PR-AUC value was variable, depending on the ML model used, and ranged from ~0.25 to ~0.5 for the best performers (GBM, RF, and XGB) using 90% of the data in the training set. Of the 27 features employed, the most important predictors of essential genes in both *C. elegans* and *D. melanogaster* were num_cells_expressed, OrthoFinder_species, and exon numbers (exons), followed by two inferred subcellular localisations (nucleus and cytoplasm). Following the prediction of essential genes in *H. contortus* using the best-performing models (GBM and XGB, trained with *C. elegans* and *D. melanogaster* features), 499 genes had a probability of >0.7 of being essential and 17,587 had a probability of <0.3. In total, >95% of the top 1000 essential genes predicted have orthologs in *C. elegans*, *D. melanogaster,* and/or *Ovis aries* (sheep host), and ≥90% are single-copy genes.

### 2.2. Clear Association between Essential Genes and Their Transcription Profiles

**Figure 2:**
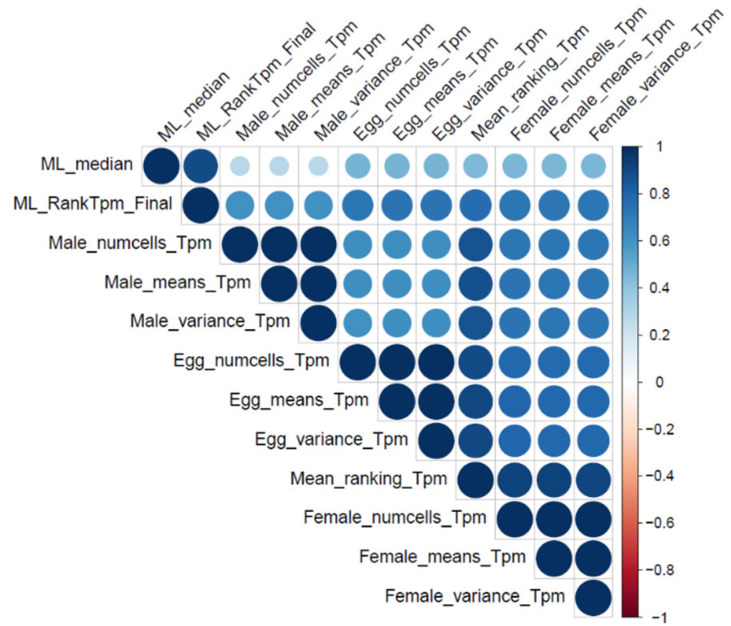
Pairwise correlations between ranked lists of genes predicted to be essential in *Haemonchus contortus* through ML and levels of transcription (snRNA-seq, number of nuclei (cells) in which a gene is transcribed, mean/average transcription level, and variance of transcription). A final score was defined by considering both ML and snRNA-seq data (ML_RankTpm_Final). Circle size represents correlation strength.

Since the feature “num_cells_expressed” was a key predictor of essentiality, we explored whether essential and non-essential genes have distinct transcription profiles. Interestingly, *H. contortus* genes ranked according to their essentiality probabilities, defined by the ML models, correlated with their rankings based on mean levels of transcription, variances, and numbers of nuclei in which these genes were transcribed (values between ~0.3 and ~0.6; Figure 2). The strongest correlation was between the ML-based ranking and transcription in the egg stage (snRNA-seq), followed by transcription in the adult female and then adult male of *H. contortus*. This order was somewhat expected since the egg snRNA-seq data were used to train the ML models and adult females contain eggs. However, the strong pairwise correlations between the different gene rankings using snRNA-seq data were unexpected. We determined a final score for each gene, taking into consideration both the ML predictions and transcription levels; the final scores for all genes correlated well (>0.7) with each ML or snRNA-seq ranking (ML_RankTpm_Final) (Figure 2). Using these final scores, we created the priority list of essential genes, the top 1000 of which are listed in Supplementary Table S1.

**Figure 3:**
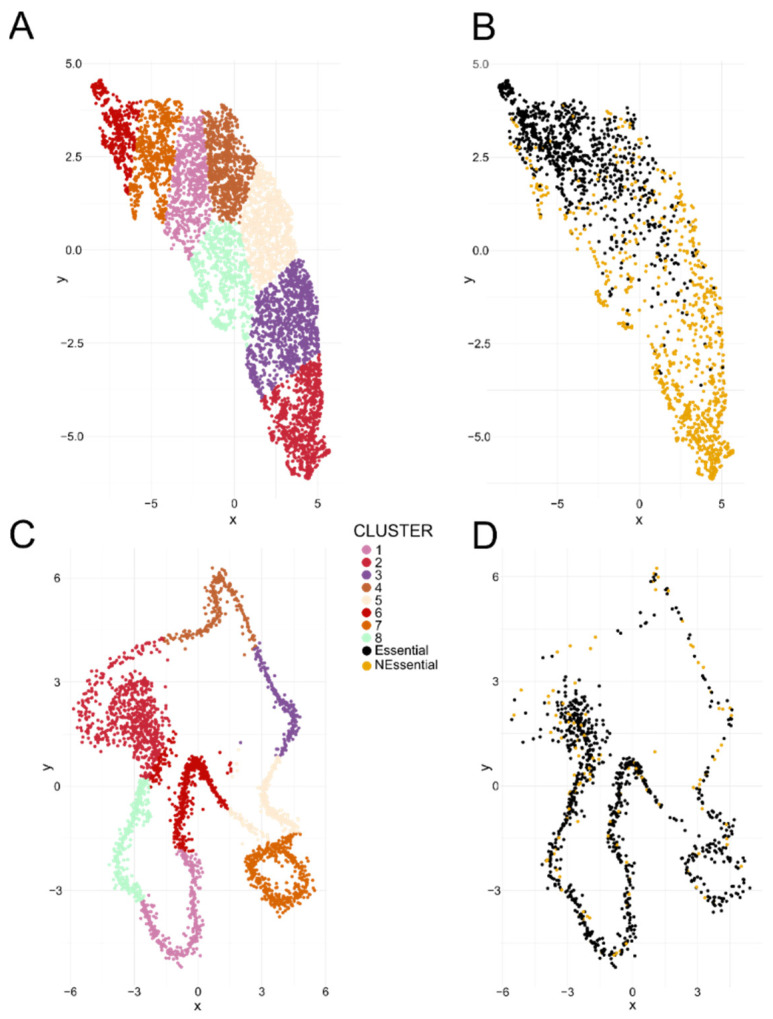
Establishing the relationship between transcription profiles and essential genes. (**A**) A selection of 6173 *Haemonchus contortus* genes was clustered using uniform manifold approximation and projection (UMAP) based on the level of transcription in 99 samples (RNA-seq; with genes transcribed in all samples included). (**B**) The same plot with 980/1000 *H. contortus* essential (black) and 905/10,000 non-essential (light orange) genes overlaid. (**C**,**D**) The clustering analysis using a *H. contortus* snRNA-seq sample (female—32,426 cells); 3000 genes with the highest mean transcription are represented. In total, 847/1000 essential (black) and 118/10,000 non-essential (light orange) genes were included.

To further explore the relationship between gene essentiality and transcription in *H. contortus*, we identified UMAP gene clusters based on their transcription (RNA-seq) in 99 distinct samples. After filtering out genes that were not transcribed in all of these samples, 6173 genes (31.7%) remained (Figure 3A). Of these genes, 980 of 1000 (98.0%) of the high-priority essential genes were present, and most of them clustered together. On the other hand, following the filtering step, only 905 of 10,000 (9.1%) of the most likely non-essential genes remained; most of these 905 genes clustered to the exclusion of the essential genes (Figure 3B). A similar observation was made following the UMAP clustering of genes linked to female snRNA-seq data; here, we used 3000 genes with the highest average transcription levels (Figure 3C,D). In this analysis, 847 of 1000 (84.7%) essential genes remained and mostly clustered together, compared with only 118 of 10,000 (1.1%) non-essential genes that remained. To confirm the grouping of essential genes based on transcription profiles, we independently evaluated data for the related species *C. elegans* using its genes and respective essentiality annotations [10]. Clusters were defined using RNA-seq data from 295 samples; following the filtering step, 833 of 1000 (83.3%) essential genes were retained and mostly clustered together whereas only 1021 of 10,000 (10.2%) of the non-essential genes remained and clustered to the exclusion of essential genes. 

### 2.3. Essential Genes of H. contortus Are Inferred to Be Involved Predominantly in Ribosome Biogenesis, Translation, RNA Binding/Processing, and Signalling

GO enrichment analysis for the prioritised list of the top 1000 essential genes of *H. contortus* inferred ribosome structure, translation, and peptide and RNA binding/processing (*p* < 10^−8^) for ‘molecular function’; peptide/amide biosynthesis and translation/gene expression for ‘biological process’ (*p* < 10^−14^); and ribosomes/ribonucleoproteins (intracellular non-membrane-bound organelles) for ‘cellular component’ (*p* < 10^−11^). Pathway enrichment analysis revealed that orthologs of many of these genes were significantly (*p* < 10^−16^) linked to functions including the following: (i) the assembly of the ribosome (e.g., GTP hydrolysis, the joining of the 60S ribosomal subunit, and the formation of free 40S subunits); (ii) translation initiation (e.g., eukaryotic and cap-dependent); and (iii) signalling and regulatory roles (e.g., L13a-mediated translation silencing of ceruloplasmin expression, SRP-dependent co-translational protein targeting to membranes, and nonsense-mediated decay).

### 2.4. Linking Essential Genes to Genome Locations and Their Transcription to Cell Type or Tissue

**Figure 4:**
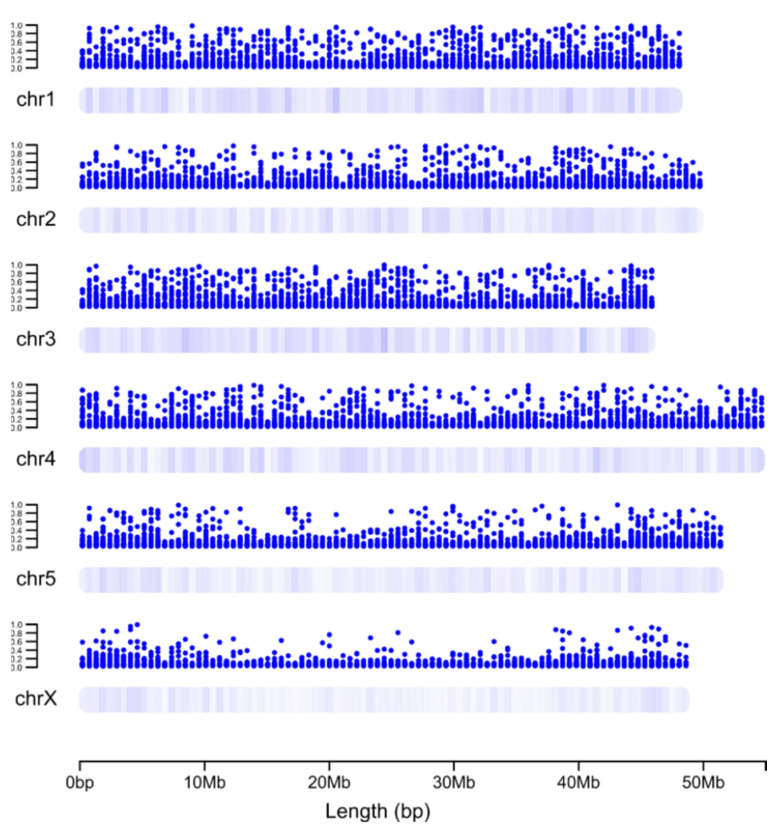
The probabilities of genes (*Y*-axis) on the autosomal (chr1-5) and sex chromosomes (chrX) of *H. contortus* being essential, as defined via machine learning (ML). The probabilities of individual genes were mapped to their respective genomic coordinates (*X*-axis).

First, we first plotted the ML-based gene essentiality probabilities along individual *H. contortus* chromosomes (Figure 4). Most (85%) of the 499 high-priority essential genes predicted (probability of >0.7) were linked to autosomal chromosomes chr1–chr4 without a distinct clustering. A small percentage (15%) of these genes were on chr5 and the sex chromosome (chrX), and were located mainly on chromosome arms. 

**Figure 5:**
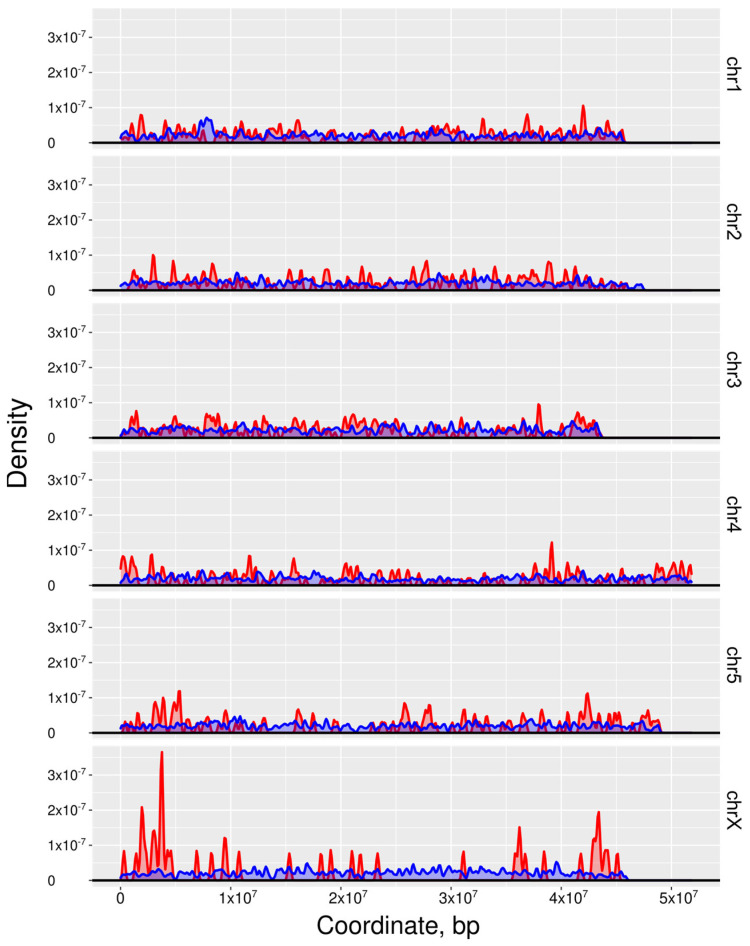
The distribution densities of both the ‘top’ 1000 genes inferred to be essential (red) as well as 10,000 non-essential (blue) genes on the autosomal (chr1-5) and sex chromosomes (chrX) of *Haemonchus contortus* as defined via machine learning (ML).

Second, we studied the density distribution of the top 1000 essential and 10,000 non-essential genes on the chromosomes (Figure 5). Essential genes were located in “hotspots” that were relatively evenly distributed on chr1–chr5, with high densities detected on the arms of chrX vis-à-vis the centre of this chromosome; there were no apparent location preferences for non-essential genes. Third, using information available for *C. elegans* (see [25]), we inferred cell types or tissues in which essential genes were highly transcribed. To do this, we mapped the transcription of *C. elegans* orthologs of the top 1000 genes predicted/prioritised as essential in *H. contortus* to known cell and tissue types in *C. elegans*. For cell types, we observed that 341 essential genes were highly transcribed in the germline, 308 in somatic gonad precursors, 209 in sex myoblasts, and 193 in vulva cell precursors. Considering only nerve cells, 193 essential genes were highly transcribed in canal-associated neurons (CANs), 174 in amphid neurons with finger-like ciliated endings (AFD), and 121 in amphid wing “A” neurons (AWA). For tissues, we identified 711 essential genes abundantly transcribed in the gonad, and 396 in glia, followed by 387 in the hypodermis.

## 3. Discussion

Extending previous work on the gene essentiality, particularly in the model Ecdysozoans *C. elegans* and *D. melanogaster* (see [17]), this study has provided the first comprehensive, large-scale prediction of essential genes in the parasitic nematode *H. contortus* using ML and included relevant complementary analyses. We have provided evidence of a relationship between essential genes and transcription and defined a feature set for *H. contortus* that will likely be a useful resource for identifying essential genes in related strongylid nematodes.

To predict gene essentiality in *H. contortus*, we used 27 features that had been shown to be strong predictors of essential genes within and between the model organisms *C. elegans* and *D. melanogaster* (see [16]), and we defined two additional features linked to sequence conservation and transcription level, which allowed reliable predictions in each of these model species. This work yielded some genomic sequence, gene conservation, and transcription profile characteristics that are key for gene essentiality predictions, corroborating some previous studies [16,17,26,27,28,29]. These types of features can be readily obtained from genomic and transcriptomic data sets, and we also inferred some highly ranked “essential genes” using features that appeared to be exclusive to *H. contortus* and that could be applied to related strongylid nematodes. Nonetheless, these predictions will need to be validated experimentally using gene knockout and/or knockdown methods (cf. [30,31]). Select features of essential genes in *C. elegans* and *D. melanogaster*, such as histone modification markers (e.g., H3K4me3 and H3K27me3; [10,11]), which have been found to be important predictors of gene essentiality, could not be assessed herein, as comparable data are not presently available for *H. contortus*. 

We identified a strong relationship between gene essentiality and transcription profile. Using snRNA-seq data, we showed that the number of nuclei for which a gene is transcribed and the level and variance of transcription correlated relatively well with ML-based predictions of gene essentiality, particularly for *H. contortus* eggs. We also showed that selections of essential genes clustered according to transcription profiles in RNA-seq and snRNA-seq data, and that “essential genes” usually grouped together to the exclusion of “non-essential” genes of *H. contortus* (Figure 3). This latter finding was supported by analyses of *C. elegans* data in that most (83.3%) “essential genes” were usually transcribed in all samples (295 bulk RNA-seq data sets), and those genes were amongst the most highly transcribed in nuclei (three snRNA-seq data sets), whereas most “non-essential genes” were lowly transcribed and, thus, were removed upon filtering. Moreover, most essential genes predicted were not present in lists of genes known to be differentially transcribed between developmental stages or sexes (cf. [21]). 

Through comparative analysis of scRNA-seq data for *C. elegans*, we inferred that a high proportion (>30–70%) of essential gene orthologs in *H. contortus* was more likely to be transcribed in tissues and cells of the reproductive tract (germline and associated tissues and cells) than other organ systems. Considering only neuronal cells, essential genes were more likely to be found in CANs than in other cell types. Interestingly, CANs are critical for the larval development and survival of *C. elegans* and are governed by regulatory mechanisms that are currently unknown [32]. These findings will pave the way for future studies of the functions, structures, and/or interactions of essential proteins encoded in these reproductive and neuronal cell types as a starting point for anthelmitic target validation. 

Essential genes were much more likely to be found on autosomes than on the sex chromosome in *H. contortus*, in accordance with findings for *C. elegans* (see [10]), although the relatively even distribution of such genes on the autosomes was distinct from that seen in *C. elegans* (in or near the centre of chromosomes; [10]) or *D. melanogaster* (away from the centre/centromeres; [11]). The distinction between *H. contortus* and *C. elegans* might relate to the different genome and/or centromere organisations and/or gene regulatory mechanisms (genetic vs. epigenetic) [33]. Despite the conservation of one-to-one orthologs between *H. contortus* and *C. elegans*, the order and location of such genes on chromosomes are distinctly different between these species [21]. Using GO and pathway analyses, we inferred that many essential genes of *H. contortus* are involved in transcriptional regulation and particularly in RNA binding, ribosome formation, and/or translation initiation functions, which supports previous findings for *C. elegans* and *D. melanogaster* (see [16,17]). Ribosome formation and translation initiation are biologically crucial and very energy-demanding (e.g., [34,35,36]), which suggests that the chemical or functional genomic disruption of these processes and associated pathways in *H. contortus* would lead to serious detrimental effects on this species. 

## 4. Materials and Methods

**Figure 6:**
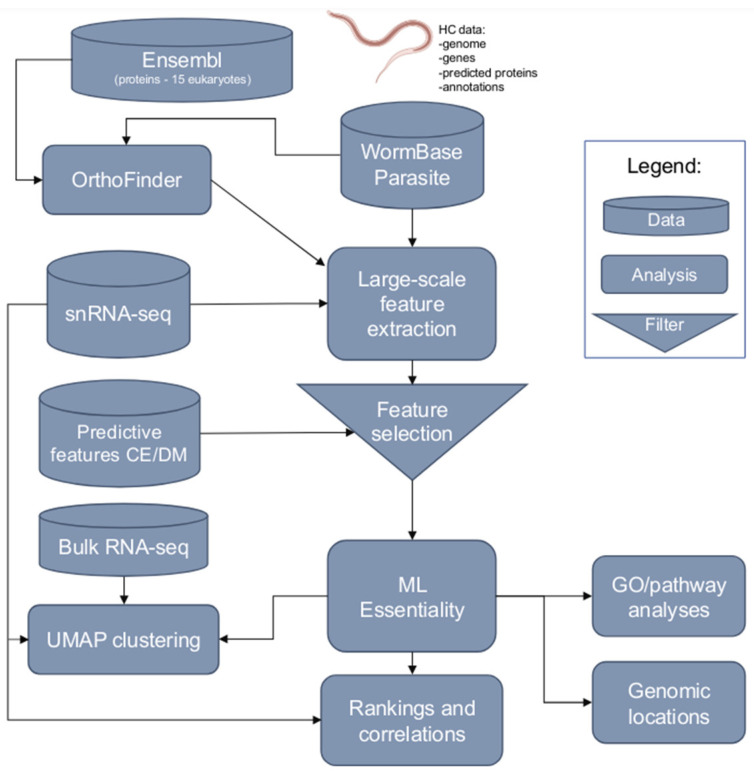
The workflow used for the prediction of essential genes in *Haemonchus contortus* using machine learning (ML) and complementary analyses. A range of features (see Materials and Methods) were extracted for *H. contortus* genes, and selected features were used to train ML models and predict essential genes. The relationship between essentiality and transcription was investigated through clustering and correlation analyses; other complementary analyses included gene ontology (GO)/pathway enrichments and genomic locations.

We obtained published genomic and transcriptomic data sets for *H. contortus* from multiple sources and employed a workflow (Figure 6) to predict essential genes and explore the transcriptional and functional characteristics of prioritised gene candidates.

### 4.1. RNA Sequence Data Sets

RNA-seq data sets from 99 *H. contortus* and 295 *C. elegans* samples (whole worms) representing different developmental stages, strains, and both sexes were obtained from WormBase Parasite [13,37]. Also, single-nuclei (sn)RNA-seq data for eggs, adult females, or adult males of *H. contortus* (Haecon5 strain) were publicly available [38]. For individual nuclei, reads that mapped to individual genes were enumerated. All data sets were normalised using transcripts per million (TPM) and loaded into data frames in R (https://www.r-project.org; accessed on 1 May 2024) for subsequent use and analyses. Genes without evidence of transcription (i.e., mapped read counts = 0) in a sample were removed. 

### 4.2. Feature Extraction/Engineering for Subsequent ML

For each gene of *H. contortus* (PRJEB506.WBPS14; WormBaseParaSite), we extracted 9588 features that were derived from RNA-seq and/or protein sequence data sets, subcellular localisation (inferred using DeepLoc 1.0; [39]), and snRNA-seq data (feature: “num_cells_expressed”—representing the number of cells/nuclei in which a particular gene is transcribed for a particular sample) using an established method [10,11]. Then, we selected 27 features for *H. contortus* (Table 1) for the prediction and evaluation of gene essentiality through ML [10]. Of these 27 features, 25 represented predictors of essential genes in both *C. elegans* and *D. melanogaster* (cf. [16]). Feature 26 was “num_cells_expressed” from *H. contortus* egg snRNA-seq data, which is a strong essential gene predictor using single-cell RNA-seq (scRNA-seq) data for early developmental stages of *C. elegans* (larval—L1; [10]) and *D. melanogaster* (embryo; [11]). Feature 27 (OrthoFinder_species) relates to protein sequence conservation among species. In brief, predicted proteomes (FASTA files) representing 16 eukaryotic species (including *H. contortus*) from divergent branches in the Tree of Life [40] were obtained from Ensembl [41] and WormBase ParaSite [13,37]; orthologous groups were identified in these proteomes using the tool OrthoFinder [42], employing default parameters. Then, we identified the number of species represented within individual orthologous protein groups, which represented feature 27 (for *C. elegans*, *D. melanogaster*, and *H. contortus*).

### 4.3. Predicting Gene Essentiality through ML

We assessed the individual and collective powers of the 27 features selected to predict essential genes in *C. elegans* and in *D. melanogaster*, employing six distinct machine learning (ML) models (Gradient Boosting Machine—GBM, Generalised Linear Model—GLM, Neural Network—NN, Random Forest—RF, Support Vector Machine—SVM, and Extreme Gradient Boosting Machine—XGB; [10,11]). The best-performing ML models for *C. elegans* and *D. melanogaster*, based on ROC-AUC and PR-AUC metrics, were used to predict essential genes in *H. contortus*. Then, corresponding orthologs in *C. elegans*, *D. melanogaster,* and *Ovis aries* (sheep host) were identified for all *H. contortus* genes using g:Profiler [43] or OrthoFinder [42].

### 4.4. Establishing the List of Genes, Ranked According to the Probability of Being Essential

We ranked all *H. contortus* genes based on their probability (descending) of being essential, defined using the best-performing ML model. Moreover, using the snRNA-seq data, three rankings per sample were established for *H. contortus* genes according to the following criteria: (i) the number of nuclei in which a particular gene was transcribed, (i) the mean transcription, and (iii) variance of transcription among all nuclei. A final ranking was established by averaging all other rankings, defined by the snRNA and ML data. To evaluate the pairwise correlations among the defined rankings, a correlation plot (“corrplot” package for R) was produced. 

### 4.5. Gene Clustering

We clustered genes according to transcription in 99 RNA-seq samples for *H. contortus* and in 295 samples representing *C. elegans*. For this analysis, we used unsupervised clustering, employing uniform manifold approximation and projection (UMAP; “umap” package for R), with random initialisation. Only genes with evidence of transcription in all RNA-seq samples were included in the analysis. Following assignment, gene clusters were displayed using “ggplot2” for R. For *H. contortus*, a similar clustering analysis was performed using snRNA-seq, representing the adult female stage, but only a selection of 3000 genes with the highest mean transcription levels among all nuclei was used.

### 4.6. Methods Used to Infer Genome Locations and Transcription Profiles for Essential Genes in H. contortus

We located the top 1000 most likely essential and 10,000 most likely non-essential genes predicted for *H. contortus* in the genome of this species using the General Feature Format (GFF) annotation file obtained from WormBase ParaSite [13,37]. The density of genes on *H. contortus* chromosomes and their probability of being essential (defined through ML) were displayed using “ggplot2” and “chromoMap” for R, respectively. We also inferred cell types and tissues in which the top 1000 “essential gene orthologs” of *H. contortus* predicted were abundantly transcribed in *C. elegans*. For these analyses, we used existing scRNA-seq data that were available (Cao_et_al_2017_vignette.RData file; cf. [25]). Genes were subjected to gene ontology (GO) and pathway enrichment analyses using g:Profiler [43] and the Reactome Knowledgebase [44], respectively.

## 5. Conclusions

In conclusion, this study provided the first genome-wide ML-based prediction and prioritisation of essential genes in *H. contortus* based on key features identified in the Ecdysozoans *C. elegans* and *D. melanogaster*. We found that the highly ranked genes in *H. contortus* were involved in fundamental molecular processes, such as transcription and translation, and exhibited transcription profiles that were distinct compared with genes that had a low ranking. The genomic locations of essential genes were quite different among the three invertebrate species, suggesting specific genomic architectures and regulation mechanisms. These findings could inform functional investigations to determine a complete set of genes that sustain life in *H. contortus*. The lack of species-specific functional genomic and histone methylation data sets for *H. contortus* was a limitation of the present study. Once available, such data would likely assist in improving ML-based predictions. Given the challenges associated with the treatment and control of *H. contortus*, it is of paramount importance to prioritise essential genes for future validation studies as drug targets by harnessing computational methods and the abundance of omic data sets available for this species. To this end, ML approaches should contribute to accelerating fundamental and applied investigations of essential genes and their suitability as drug targets, enabling the development of novel anthelmintics. The approach employed here can be extended to explore essential genes in other parasitic worms.

## Figures and Tables

**Table 1:** Features (*n* = 27) that were used to predict essential genes in *Haemonchus contortus*; these features are predictive of essential genes within and between *Caenorhabditis elegans* and *Drosophila melanogaster*.

Feature	Description	Source
OrthoFinder_species	Orthologs in other species	OrthoFinder analysis
num_cells_expressed	Number of cells/nuclei where a gene is transcribed	snRNA-seq data
exons	Number of exons	BioMart (WomBase ParaSite)
exons_total_length	Total length of exons	BioMart (WomBase ParaSite)
Cytoplasm	Subcellular localisation	DeepLoc analysis
Mitochondrion	Subcellular localisation	DeepLoc analysis
Nucleus	Subcellular localisation	DeepLoc analysis
AAC_S	Protein sequence feature	Extracted using protR *
APAAC_Pc2.Hydrophobicity.2	Protein sequence feature	Extracted using protR *
CTDC_secondarystruct.Group1	Protein sequence feature	Extracted using protR *
CTDD_prop4.G2.residue0	Protein sequence feature	Extracted using protR *
CTDD_prop4.G2.residue25	Protein sequence feature	Extracted using protR *
CTriad_VS153	Protein sequence feature	Extracted using protR *
CTriad_VS431	Protein sequence feature	Extracted using protR *
CTriad_VS613	Protein sequence feature	Extracted using protR *
DC_HA	Protein sequence feature	Extracted using protR *
DC_MP	Protein sequence feature	Extracted using protR *
DC_MS	Protein sequence feature	Extracted using protR *
DC_VF	Protein sequence feature	Extracted using protR *
Geary_CHOC760101.lag7	Protein sequence feature	Extracted using protR *
Moran_CHAM820102.lag7	Protein sequence feature	Extracted using protR *
GC	DNA sequence feature	BioMart (WormBase ParaSite)
kmer_3_GCT	DNA sequence feature	Extracted using rDNAse *
PseKNC_3_Xc1.CCC	DNA sequence feature	Extracted using rDNAse *
PseKNC_5_Xc1.CGT	DNA sequence feature	Extracted using rDNAse *
PseKNC_5_Xc1.GCT	DNA sequence feature	Extracted using rDNAse *
TACC_Nucleosome.lag2	DNA Sequence feature	Extracted using rDNAse *

* For further information about those sequence features, refer to the documentations of the R packages protR (https://cran.r-project.org/web/packages/protr/vignettes/protr.html, accessed on 1 May 2024) and rDNAse (https://cran.r-project.org/web/packages/rDNAse/vignettes/rDNAse.pdf, accessed on 1 May 2024).

## Data Availability

The data and code used are publicly available and have been referenced in the Materials and Methods section.

## Supplementary Materials

The following supporting information can be downloaded at: https://www.mdpi.com/article/10.3390/ijms25137015/s1.
